# Moderate rate of implementation of spinal anesthesia for cesarean section: does it improve neonatal well-being? A case–control study

**DOI:** 10.1038/s41598-020-80666-7

**Published:** 2021-01-08

**Authors:** Yuki Sugo, Mirei Kubota, Hidetomo Niwa, Kazuyoshi Hirota

**Affiliations:** 1 Department of Anesthesiology, Hirosaki University Hospital, 53 Honcho, Hirosaki, Aomori 036-8563 Japan; 2 Department of Anesthesiology, Hirosaki University Graduate School of Medicine, 5 Zaifucho, Hirosaki, Aomori 036-8562 Japan

**Keywords:** Paediatrics, Outcomes research, Paediatric research

## Abstract

Before 2013, almost none of the cesarean section (CS) deliveries at our institution were performed with spinal anesthesia (SA), but after 2013 SA became the first-choice anesthesia for CS because it achieved better neonatal outcomes. However, the current rate of SA implementation at our institution was estimated to be approx. 60–70%, which is intermediate between these at other institutions in Japan or in other countries. This raises a question: What rate of SA use among CS cases achieves the best neonatal outcomes? To answer this question, we conducted this single-center case–control study with 1326 CS cases between 1994 and 2017 and compared the neonatal outcomes before to those after 2013. The logistic regression models were prepared to estimate the risk of birth asphyxia defined as a 5-min Apgar of < 7, associated with eight potential confounders, including the modified anesthetic protocol. The modified protocol was not a significant independent factor for neonatal asphyxia, indicating that our moderate SA priority protocol did not improve the neonatal outcomes even when compared to the outcomes at a 0% SA rate. A > 70% rate of SA implementation may be needed to provide better neonatal outcomes.

## Introduction

It has been globally accepted that spinal anesthesia (SA) is the first-line anesthesia for deliveries by cesarean section (CS), since SA provides a better neonatal outcome as well as maternal safety^1,2^. General anesthesia (GA) is thus used in a very limited number of CS cases and is typically indicated for emergent/urgent cases or for patients with contraindications for neuraxial anesthesia.

At our institution, almost 100% of the cesarean cases performed since the 1960s were done with GA due to our institutional policy, but in 2013 we adopted SA as the first-line anesthesia in cesarean cases in accordance with globally accepted obstetric practice guidelines. We have thus given priority to SA for CS cases since 2013. However, we speculated that the actual rate of SA usage at our institution after 2013 was around 60% to 70%. In contrast, an analysis of recent obstetric anesthetic practice in the U.S. using National Anesthesia Clinical Outcomes Registry data (n = 218,285 CS cases) showed that the rate of SA usage was 94.2% of all CS cases and 85.4% of emergent CS cases^3^. In Japan, a nationwide population-based study using obstetric data from 2010 to 2013 revealed that the proportion of SA usage in elective CS cases was 88.7%^1^. We thus realized that the rate of SA usage for CS at our institution was still “moderate,” considering that SA usage at other institutions is approximately 90%.

This led us to ask the following question: "How often should anesthesiologists perform SA in order to contribute to better neonatal outcomes when managing CS delivery?” Put another way: "Did this moderate rate of SA implementation at our institution improve neonatal outcomes?".

To answer these questions, we conducted the present retrospective case–control study using the obstetric data and compared the neonatal outcomes before to those after the CS anesthetic protocol was modified.

## Results and discussion

A total of 1350 patients treated during the period from 1994 to 2017 were included in the analyses. Among them, 24 patients were excluded due to incomplete data; thus, 1326 cases were included in the final analysis.

### Review of the 2013–2017 obstetric data

After the anesthetic protocol was modified, 413 CS cases were performed. Among the 413 patients, 287 (69.5%) received SA and 126 (30.5%) received GA. The rates of SA use for each type of surgery are shown in Table 1: these rates were 78% in the elective cases, 61.4% in the emergent cases, and 30.4% in the urgent cases.

**Table 1:** GA and SA after the modified protocol.

	Elective	Emergent	Urgent
Spinal anesthesia, n (%)	191 (78)	89 (61.4)	7 (30.4)
General anesthesia, n (%)	54 (22)	56 (38.6)	16 (69.6)
Total, n	245	145	23
**Reason for GA**			
CTG grade over 3, n (%)	0 (0)	20 (35.7)	11 (68.8)
Obstetric complication, n (%)	40 (74.1)	48 (85.7)	15 (93.8)
Preg over 35 years, n (%)	11 (20.4)	16 (28.6)	5 (31.3)
Tocolytic therapy, n (%)	1 (1.9)	8 (14.3)	3 (18.8)
Non-full term, n (%)	2 (3.7)	27 (48.3)	3 (18.8)
PROM, n (%)	0 (0)	13 (23.2)	2 (12.5)
IUGR, n (%)	1 (1.9)	4 (7.1)	0 (0)
Congenital anomaly, n (%)	4 (7.4)	2 (3.6)	0 (0)
Meconium staining, n (%)	0 (0)	14 (25)	5 (31.3)
Malpresentation, n (%)	7 (13)	9 (16.1)	3(18.8)
Birthweight < 1500, n (%)	0 (0)	4 (7.1)	0 (0)

GA, General anesthesia; SA, Spinal anesthesia; n, Number; CTG, Cardiotocography; GA, General anesthesia; IUGR, Intrauterine growth retardation; Peg, Pregnancy; PROM, Premature rupture of membrane.

Among the patients who received GA for elective CS, there were no cases with a fetal cardiotocography grade over 3. Even in the emergent surgery group, cases with a fetal cardiotocography grade over 3 accounted for only 35.7% of cases. In contrast, almost 70% of the urgent cases had fetal bradycardia, and > 90% had obstetric complications.

### The effect of the modified anesthetic protocol on the neonatal outcomes

#### Patient characteristics: all CS cases in 1994–2017

The characteristics of all 1326 cesarean sections performed during the study period (i.e., 1994–2017) are summarized in Table 2. Compared to the patients after 2013 (i.e., when the anesthesia protocol was modified), those who underwent a CS before 2013 were significantly older and were at earlier gestational ages (i.e., non-full term). The proportions of emergent surgeries and of neonates with birthweights ≤ 1500 g were significantly higher before 2013. In contrast, the proportion of patients with obstetric complications was significantly lower before 2013. The incidence of neonatal asphyxia after 2013 was significantly lower at 1 min but not at 5 min.

**Table 2:** The characteristics of all cesarean section cases between 1994 and 2017.

	After modif n = 413	Before modif n = 913	MD (95%CI)	OR (95%CI)	*p *value
**Age, n (%)**			n.a	n.a	< 0.001
≤ 19 years	1 (0.2)	4 (0.4)			
20–34 years	205 (49.6)	308 (33.7)			
35–39 years	155 (37.5)	512 (56.1)			
≥ 40 years	52 (12.6)	89 (9.7)			
**BMI, n (%)**			n.a	n.a	0.005
< 24	185 (45.9)	506 (55.5)			
25–29	169 (41.9)	312 (34.2)			
≥ 30	49 (12.2)	93 (10.2)			
Gestational age, weeks	37.4 ± 2.0	33.6 ± 5.2	− 3.84 (− 4.36, − 3.32)	n.a	< 0.001
Non full-term, n (%)	76 (18.4)	642 (70.3)	n.a	0.10 (0.07,0.13)	< 0.001
Obstetric complications, n (%)	310 (75.1)	296 (29.6)	n.a	7.17 (5.51, 9.34)	< 0.001
Emergency, n (%)	168 (40.7)	534 (59.0)	n.a	0.48 (0.38, 0.60)	< 0.001
General anesthesia, n (%)	126 (30.5)	911 (99.8)	n.a	0.001 (0, 0.004)	< 0.001
Birthweight < 1500 g, n (%)	8 (1.9)	91 (10.0)	n.a	0.18 (0.09, 0.37)	< 0.001
Cord blood pH < 7.2, n (%)	10 (2.5)	22 (2.9)	n.a	0.86 (0.40, 1.83)	0.698
1-min APS < 7, n (%)	61 (14.8)	212 (23.2)	n.a	0.57 (0.42, 0.78)	< 0.001
5-min APS < 7, n (%)	29 (7)	83 (9.1)	n.a	0.75 (0.49, 1.17)	0.207

Modif, The protocol was modified; APS, Apgar score; BMI, Body mass index; MD, Mean difference; n.a., Not analyzed; OR, Odds ratio.

#### A case–control study

##### Logistic regression analysis of the primary outcome

No linearity was observed for any of the quantitative variables, but among the four possible confounders (the anesthetic protocol, maternal obstetric complications, type of surgery, and GA usage), the GA usage had significant multicollinearity with the modified anesthesia protocol (Spearman's rank correlation coefficient r_s_ = 0.78). This result indicated that the modified anesthetic protocol was significantly associated with the rate of GA usage, since the rate of GA usage was almost 100% before the protocol was modified at our institution but it clearly decreased thereafter. Therefore, inclusion of the GA usage in this regression analysis would have interfered with the inverse matrix calculations, resulting in low accuracy. We thus excluded general anesthesia as a variable in the subsequent analyses.

The results of the logistic regression analysis are given in Table 3. The *p*-values of six variables (modified protocol, maternal age, gestational age, emergent surgery, neonatal weight, and cord blood pH) were < 0.25 in the univariate analysis. We thus used these six variables in the multivariate analysis. The Hosmer–Lemeshow test showed that the fit for the logistic model was good (*p* = 0.403). The percentage of correct classifications was also good (93.1%). No outliers (defined as a prediction value ≥ the measured value ± 3 SD) were identified. When the six variables in the prediction model were allowed to vary independently, 82.7% of the cross-validated grouped cases were correctly classified.

**Table 3:** Univariate and multivariate analysis of risk for APS at 5 min < 7.

	Univariate analysis			Multivariate analysis	
	OR (95%CI)	Effect size	*p *value	OR (95%CI)	*p *value
Modified protocol	0.75 (0.49–1.17)	− 0.035	0.206	1.56 (0.86–2.85)	0.145
Age	0.74 (0.62–0.89)	0.106	0.002	0.77 (0.60–0.97)	0.026
BMI	0.80 (0.59–1.08)	0.042	0.312	n.a.	n.a.
Non full-term	3.22 (2.03–5.09)	0.144	< 0.001	4.55 (2.36–8.77)	< 0.001
Obstetric complications	1.08 (0.73–1.59)	0.010	0.704	n.a.	n.a.
Emergent surgery	3.02 (1.93–4.72)	0.139	< 0.001	1.49 (0.85–2.60)	0.163
Birthweight < 1500 g	7.82 (4.86–12.57)	0.267	< 0.001	3.61 (1.76–7.41)	< 0.001
Cord blood pH < 7.2	5.78 (2.58–12.95)	0.141	< 0.001	7.39 (3.00–18.18)	< 0.001

APS, Apgar score; min, Minutes; BMI, Body mass index; CI, Confidence interval; n.a., Not analyzed; OR, Odds ratio; Effect size: Cramer’s *V.*

The modified protocol variable was not a significant independent factor for neonatal asphyxia (*p* = 0.145). Emergent surgery was also not significant. In contrast, the other four variables (maternal age, gestational age, neonatal birthweight, and cord blood pH) were significant independent factors.

##### Logistic regression analysis of the secondary outcome

We performed a multivariate analysis using the seven variables in the logistic regression analysis of the secondary outcome (i.e., the incidence of neonatal asphyxia at 1 min after birth). The Hosmer–Lemeshow test showed that the fit for the logistic model was good (*p* = 0.780). The percentage of correct classifications was also good (84.0%). No outliers (defined as a prediction value ≥ the measured value ± 3 SD) were detected. When the seven variables in the prediction model were allowed to vary independently, 78.6% of cross-validated grouped cases was correctly classified. The modified anesthesia protocol variable was not a significant independent factor for neonatal asphyxia (*p* = 0.999) (Table 4).

**Table 4:** Univariate and multivariate analysis of risk for APS at 1 min < 7.

	Univariate analysis			Multivariate analysis	
	OR (95%CI)	Effect size	*p *value	OR (95%CI)	*p *value
Modified protocol	0.57 (0.42–0.78)	− 0.097	< 0.001	1.00 (0.64–1.57)	0.999
Age	0.79 (0.70–0.90)	0.129	< 0.001	0.86 (0.73–1.00)	0.054
BMI	0.90 (0.74–1.10)	0.031	0.542	n.a.	n.a.
Non full-term	2.82 (2.11–3.79)	0.195	< 0.001	2.53 (1.70–3.76)	< 0.001
Obstetric complications	1.20 (0.92–1.57)	0.037	0.176	1.20 (0.83–1.73)	0.341
Emergent surgery	2.84 (2.12–3.80)	0.198	< 0.001	1.69 (1.18–2.40)	0.004
Birthweight < 1500 g	11.52 (7.34–18.15)	0.345	< 0.001	7.52 (3.93–14.41)	< 0.001
Cord blood < 7.12	4.83 (2.38–9.83)	0.140	< 0.001	6.02 (2.77–13.06)	< 0.001

APS, Apgar score; min, Minutes; BMI, Body mass index; CI, Confidence interval; n.a., Not analyzed; OR, Odds ratio; Effect size: Cramer’s *V.*

We conducted a retrospective case–control study to clarify the impact of the modified anesthetic protocol for CS at our institution on neonatal outcomes. This protocol gives priority to SA. Our findings demonstrated that the rate of SA usage for CS at our institution was approximately 70%. Given that the rate of SA usage in other institutions is approximately 90%^1,3^, we considered that the observed rate of implementation of SA at our institution was “moderate”. The results of our analyses also revealed that this moderate prioritization of SA did not improve the neonatal outcomes compared to a strict prioritization of GA (SA 0%), as the modified protocol was not associated with the incidence of neonatal asphyxia at either 1 or 5 min after birth (1-min APS < 7, OR: 1.00, 95%CI: 0.64–1.57, *p* = 0.999; 5-min APS < 7, OR: 1.56, 95%CI: 0.86–2.85, *p* = 0.145).

Before 2013, almost none of the patients who underwent a cesarean section at our institution received SA, but after 2013, the rate of SA usage was dramatically increased from 0 to 70% of all CS deliveries. We assumed that such a dramatically increased rate of SA usage would be associated with an improvement in neonatal outcomes, since SA has been reported to be ranked best for APS in CS, among the available anesthetic techniques^1,2^. However, the results of the present analyses showed that there was no significant difference in the incidence of neonatal asphyxia between a 70% SA-adoption rate for CS deliveries and a 0% SA-adoption rate for CS deliveries. The rate of SA use has been reported to be strict in the U.S. (i.e., 94.2% of all CS cases)^3^ as well as in Japan (i.e., 88.7% of elective CS cases)^1^. The perinatal registration database of the Japan Society of Obstetrics and Gynecology showed that the rate of neonatal asphyxia with 5-min APS of < 7 in 2018 was 2.4% of all deliveries. A retrospective analysis of institutional data from Finland showed that the rate of neonatal asphyxia with 5-min APS of < 7 was 0.4–0.6% in CS cases^4^. A population-based study in Australia also showed that the incidence of neonatal asphyxia in CS (5-min APS < 7) was low at 1% with 83.2% of SA usage^5^. In contrast, the rate of neonatal asphyxia at our institution was higher at 7% with lower SA usage at 70%. These findings indicate that “strict” (i.e., > 90% or > 80%) SA performance may be needed to improve neonatal outcomes.

The lower rate of SA usage at our institution may have been due to a high rate of maternal indications for GA in the elective as well as emergent cases. Among the present group of elective GA cases, we observed no cases with a CTG grade over 3, and only 35.7% of the emergent GA cases had a CTG grade over 3. We therefore speculate that the use of a stricter set of SA/GA indications can significantly decrease the incidence of neonatal asphyxia.

SA is advantageous for neonatal well-being. However, we should consider the limits of SA that cannot be prolonged during surgery. In the case of bladder injuries during the CS, GA should be considered. Franchi et al. reported that among 28,822 deliveries, 7616 were CS cases (26.4%) and 3 cases of unintentional transvesical (UTV)-CS, defined as any extraction of the fetus through a double full thickness bladder wall cystotomy, were identified (incidence rate: 0.039%)^6^. Considering this previous report on the incidence of UTV-CS^6^, and extrapolating the perinatal registration data base of Japan, we also had estimated 3 UTV-CS cases in 2018. We did not collect the data on UTV-CS in the present study. The effect of this uncollected data on the results is unknown.

Some limitations of this study should be acknowledged. First, this study had a single-center, retrospective design. Thus, the external validity of the present prediction model was not assessed. Second, we conducted a multivariate logistic regression analysis for risk adjustment. However, unknown or unmeasured confounders might exist and could lead to a residual bias. We could not collect the detailed obstetric data such as the CTG grade, IUGR, congenital anomaly, and obstetric complications before 2013, while those were obtained after 2013. Such detailed maternal and fetal data may have been identified additional confounders in this study. In addition, maternal background pathologies can also be possible confounders, since these factors can affect the neonatal outcomes as well as the choice of anesthesia for CS. We collected obstetric complications such as preeclampsia, pregnancy induced hypertension, gestational diabetes mellitus^7^, placenta previa, and placental abruption during the study period. However, we did not collect the data on endometriosis. This must be considered an unmeasured confounder in the present study, since endometriosis is an estrogen-dependent disease that affects women in reproductive age^8,9^ and is correlated with a risk of hypertension, placenta previa, and low newborn weight. We should consider investigating this point in a future paper. Third, almost 100% of the CS patients at our institution received GA before 2013. Such completely homogenous data might have influenced the results. However, we were able to use the data to compare the neonatal outcomes of strict GA usage (0% SA) with that of “moderate” SA usage (70% SA), and this could be considered a strong point of this study. Fourth, we evaluated the neonatal outcomes using the 1-min and 5-min APS values, but the predictive value of a low APS for poor neonatal outcome can be controversial. Low APS has been reported to be no evidence of asphyxia^2^. In contrast, it has been reported that the “traditional” low APS defined as < 7 remains relevant for the prediction of neonatal adverse outcomes^10,11^. Some significant studies showed that APS at 5 min was a prognostic factor of neonatal adverse outcomes^2,10,12^ and indeed chose APS at 5 min as a measure to evaluate neonatal outcomes^5,13^. In a joint statement issued by the Japan Society of Obstetrics and Gynecology and the Japan Association of Obstetricians and Gynecologists, neonatal asphyxia was defined as a case with 5-min APS of < 7. However, asphyxia was defined as a cord umbilical arterial pH value < 7.20 as well^14^. We thus should have evaluated the risk of neonatal asphyxia defined as a cord umbilical arterial pH value < 7.20 as well; however, it was not possible to evaluate this due to the underpowered number of neonates with a cord pH value of 7.2 in this study (n = 32). Finally, we tried to answer the question “How often should anesthesiologists perform SA for better neonatal outcomes?” In answer, we found that a rate of more than 80–90% SA use is needed. However, this answer assumes that the incidence of neonatal asphyxia is low at institutions with a high rate of SA implementation, and also assumes that SA is advantageous for neonatal well-being. The effect of a high rate of SA use on neonatal outcomes must be investigated in the future.

In conclusion, SA is considered the first-line anesthesia for cesarean sections due to the better neonatal outcomes it provides. However, the results of this retrospective case–control study demonstrated that an approx. 70% rate of SA implementation did not improve the neonatal outcomes compared to a 0% rate of SA implementation. Because other institutions with higher rates of SA implementation have reported better neonatal outcomes than ours, an 80–90% rate of SA use may be needed to contribute to better neonatal outcomes of cesarean sections.

## Methods

### Patient data collection

We obtained the patients' data from the obstetric and anesthetic charts of cases of cesarean sections performed at our institution between 1994 and 2017.

### Review of obstetric characteristics after 2013

We first reviewed the obstetric and anesthetic characteristics of the CS cases managed after the anesthetic protocol was modified in 2013 in order to determine how often SA was administered. The collected data were as follows: maternal age, body mass index (BMI), gestational age, type of surgery (elective, emergent, urgent) and anesthesia (spinal or general anesthesia), the number of patients who received tocolytic therapy, premature rupture of the membrane, and obstetric complications as well as the neonatal birthweight, umbilical cord blood pH, Apgar score (APS) at 1 and 5 min, cardiotocography (CTG) grade, the number of congenital anomalies, intrauterine growth retardation, malpresentation, and meconium staining. An APS < 7 was defined as neonatal asphyxia^15^, and abnormal umbilical cord blood pH was defined as < 7.2^2^.

### A case–control study

#### The definition of “cases” and “controls”

In accordance with the STROBE statement for observational study^16^, we considered the present retrospective study as a case–control study. We defined cases as CS deliveries performed after 2013, when the CS anesthetic protocol was modified at our institution. In contrast, controls were the CS deliveries performed before 2013. The primary neonatal outcome was the infant's well-being evaluated by the incidence of neonatal asphyxia at 5 min after birth, defined as a 5-min APS of < 7. The secondary outcome was the incidence of neonatal asphyxia at 1 min after birth, defined as a 1-min APS of < 7. We conducted the present case–control study using a logistic regression analysis. The dependent variable was the primary and secondary outcomes described above (i.e., neonatal asphyxia defined as a case with APS at 1 and 5 min < 7). The details of the analysis are described below.

#### The definition of neonatal asphyxia

In the present study, neonatal asphyxia was defined as a case with 5-min APS of < 7. A low APS has been reported to be defined as < 7, and has been associated with adverse clinical outcomes^2,10^. In an analysis of the data for 113,300 preterm infants from the Swedish Medical Birth Register, APS at 5 and 10 min provided prognostic information about neonatal survival^12^. In a prospective study investigating the effect of anesthetic technique (GA vs. SA) on neonatal morbidity in emergency CS for fetal distress, the neonatal morbidity was defined as a 5-min APS < 7, need for mechanical ventilation, admittance to a neonatal intensive care unit, or respiratory insufficiency symptoms^13^.

#### The logistic regression analysis

We investigated whether the modified protocol (i.e., using SA as the first-line anesthesia) affected the fetal outcomes at our institution by performing a logistic regression analysis of the clinical data obtained in the CS cases at our institution from 1994 to 2017. The collected data were as follows: maternal age, BMI, gestational age, obstetric complications, type of surgery (elective, emergent), anesthesia (spinal or general anesthesia), neonatal birthweight, umbilical cord blood pH, and APS at 1 and 5 min.

We prepared logistic regression models to estimate the risk of neonatal asphyxia associated with potential confounders, including clinical variables such as the modified anesthetic protocol, maternal age, BMI, gestational age, obstetric complications, type of surgery (elective or emergent), use of general anesthesia, neonatal birthweight, and umbilical cord blood pH. The inclusion of variables in the models was based on the current clinical knowledge of risk factors for neonatal asphyxia. We converted the quantitative variables (e.g., maternal and gestational age, BMI, neonatal body weight, and umbilical cord pH) into categorical variables as follows: maternal age (years old: ≤ 19 = 1, 20–34 = 0, 35–39 = 2, ≥ 40 = 3), gestational age (weeks: 37–41 = 0 as full-term, others = 1 as non-full-term), BMI (≤ 24 = 0, 25–29 = 1, ≥ 30 = 2), neonatal birthweight (> 1500 g = 0, ≤ 1500 g = 1), umbilical cord pH (> 7.2 = 0, ≤ 7.2 = 1).

Other categorical variables such as the anesthetic protocol, type of surgery, maternal obstetric complications, and use of GA were coded as follows: anesthetic protocol (patients before = 0, patients after the protocol was modified = 1), type of surgery (elective = 0, emergency = 1), maternal complications (without = 0, with = 1), GA usage (no = 0, yes = 1). The linearity of the dependent variable (i.e., the APS at 5 min) for neonatal asphyxia in the logit of the independent variable was assessed using locally weighted least squares smoothing curves. This analysis was conducted to confirm that binomial logistic regression was the appropriate method of analysis.

As a first step, we performed a univariate logistic regression analysis between the neonatal outcomes and each potential independent variable. Pearson's product-moment correlation coefficient or Spearman's rank correlation coefficient was calculated to check for multicollinearity of categorical variables. The scatter plot was also used to check for multicollinearity of quantitative variables. The probability (*p*)-levels for the model used a variable entry criterion of 0.25. The variables with *p*-values < 0.25 were thus analyzed in the next step (i.e., the multivariate analysis).

We used multivariate logistic regression models with the direct method. We estimated a logistic model which contained all covariates as possible confounders. None of the interaction terms were included in the multivariate model. The goodness of fit for the logistic model was assessed with the Hosmer–Lemeshow test. Data with missing values were excluded from the logistic regression analysis with commonly used statistical software. To examine the degree of overfitting of the prediction model to the development sample, we performed a cross validation procedure.

### Statistical analysis

For continuous variables with a normal distribution, the mean (± standard deviation [SD] or standard error [SE]) is reported. For variables not normally distributed, the median and interquartile ranges are reported. Categorical variables are presented as numbers and percentages. *P*-values < 0.05 were considered significant. The χ^2^ test was used for the analysis of categorical data, and Student's t-test was used for continuous variables with normal distributions. The Mann–Whitney rank-sum test was used for continuous variables without a normal distribution. The 95% confidence interval (CI) was computed around the mean difference in two values or the odds ratio (OR).

In the logistic regression analysis of the obstetric data obtained in the period 1994–2017, we followed standard methods to estimate the sample size that is necessary for a multivariate logistic regression, with at least ten outcomes needed for each included independent variable. With an expected neonatal asphyxia at 5 min incidence rate of 7%, we required 1000 patients to appropriately perform the multivariate logistic regression with seven variables. All statistical analyses were conducted with IBM SPSS statistics ver. 22.0 software (IBM, Tokyo, https://www.ibm.com/support/pages/downloading-ibm-spss-statistics-22).

### Ethical approval and informed consent

This single-center, retrospective study was carried out at Hirosaki University Hospital, Hirosaki, Japan. The study was approved by the Hirosaki University Graduate School of Medicine Institutional Review Board (rinri@hirosaki-u.ac.jp, approval no. 2014-079, data of approval: June 11th, 2014) before it began, and written informed consent was obtained from all patients participating in the study. If subjects were under 18, written informed consent was provided from a parent or legal guardian. All experiments were performed in accordance with relevant guidelines and regulations.

## Abbreviations

APS: Apgar score
BMI: Body mass index
CS: Cesarean section
GA: General anesthesia
SA: Spinal anesthesia
